# Dairy *Propionibacterium* extends the mean lifespan of *Caenorhabditis elegans* via activation of the innate immune system

**DOI:** 10.1038/srep31713

**Published:** 2016-08-17

**Authors:** Gayeung Kwon, Jiyun Lee, Young-Hee Lim

**Affiliations:** 1 Department of Public Health Science (Brain Korea 21 PLUS program), Graduate School, Korea University, Seoul 136-701, Republic of Korea; 2 School of Biosystem and Biomedical Science, College of Health Science, Korea University, Seoul 136-701, Republic of Korea; 3 Department of Laboratory Medicine, Korea University Guro Hospital, Seoul 152-703, Republic of Korea

## Abstract

Dairy *Propionibacterium freudenreichii* is a candidate non-lactic acid probiotic. However, little information is available on the effect of *P. freudenreichii* on lifespan extension in humans. The aim of this study was to evaluate the effects of *P. freudenreichii* on lifespan extension and to elucidate the mechanism of *P. freudenreichii*-dependent lifespan extension in *Caenorhabditis elegans*. The results showed that *P. freudenreichii* significantly (*p* < 0.05) extended the lifespan of *C. elegans* compared with *Escherichia coli* OP50, a standard food for the worm. Analysis of age-related biomarkers showed that *P. freudenreichii* retards ageing. Moreover, *P. freudenreichii* increased resistance against a human pathogen, *Salmonella typhimurium*, through the activation of *skn-1*, which is involved in pathogen resistance in *C. elegans*. Furthermore, *P. freudenreichii*-fed *daf*-*16, jnk-1, skn-1* or *daf-7* loss-of-function mutants showed an extended mean lifespan compared with *E. coli* OP50-fed worms. However, the increase in lifespan was not observed in *pmk-1, sek-1, mek-1, dbl-1, daf-12* or *daf-2* mutants, which suggests potential roles for these genes in *P. freudenreichii*-induced longevity in *C. elegans*. In conclusion, *P. freudenreichii* extends the lifespan of *C. elegans* via the p38 MAPK pathway involved in stress response and the TGF-β pathways associated with anti-inflammation processes in the immune system.

Probiotics play an important role in host intestinal health and directly and/or indirectly regulate intestinal microflora. Probiotics are effective in decreasing the colonization of pathogenic bacteria in the intestines, increasing the immune response and regulating mucosal immune signalling^1^,^2^. *Propionibacterium freudenreichii* is a candidate non-lactic acid probiotic^3^. *P. freudenreichii* has been used in the fermentation of Swiss Emmental cheese and is found in dairy products, such as milk and cheese. The short-chain fatty acid (SCFA)-producing bacterium, *P. freudenreichii,* predominantly ferments lactate to acetate, propionate and carbon dioxide. Acetate and propionate have been shown to enhance human gut immunity^4^. Recently, the benefits of *P. freudenreichii* intake, such as modulating immunity, reducing inflammation and protecting against pathogens, have been reported^5^. *P. freudenreichii* also functions as an immunomodulator of the human immune system^6^.

*Caenorhabditis elegans* is a small, free-living soil nematode commonly used as a model experimental animal because it is easy to treat, has a short lifespan, can be safely used in the laboratory and propagates through self-fertilization^7^. In particular, *C. elegans* is frequently used in studies on longevity, immunity, neurodegenerative diseases, fat storage, DNA damage responses and apoptosis^8^. *C. elegans* is a good research model because two-thirds of the genes related to human diseases are conserved in the nematode^8^,^9^,^10^. Several human pathogens also infect the intestine of *C. elegans*; thus, immune response against pathogens can be studied in *C. elegans*^11^. The immune system of *C. elegans* has evolved into the highly complex immunity of vertebrates. The relatively simple immunity of *C. elegans* might be experimentally advantageous in studying the immune signalling pathways. Notably, *C. elegans* research has elucidated entire genomes and structures of immune genes; therefore, many studies have used nematodes for studying the immune response^12^. Ageing and immunity are closely connected in *C. elegans*^13^. The *C. elegans* immune system is regulated by three major pathways of the defence system against pathogens: the transforming growth factor-beta (TGF-β) pathway, the p38 mitogen-activated protein kinase (MAPK) pathway and the Daf-2/DAF-16 pathway^14^. In the DBL/TGF-β pathway, following the binding of the TGF-β-like ligand encoded by the *dbl-1* gene to TGF-β receptors in cell membranes, SMAD proteins (SMA-2, SMA-3 and SMA-4) are phosphorylated and activate TGF-β target genes related to antibacterial defence. In the MAPK pathway, SEK-1 and MEK-1 activate PMK-1, which is involved in innate immunity in *C. elegans*, but the downstream signalling of PMK-1 has not been elucidated. The DAF-2/DAF-16 pathway is involved in antibacterial defence and regulates lifespan in *C. elegans*. DAF-16 increases stress resistance in *C. elegans* in response to insulin/insulin-like growth factor-1 (IGF-1) signalling (IIS), which plays an important role in ageing. In *C. elegans*, the IIS receptor DAF-2 inhibits the transcription factor DAF-16; thus, decreased IIS increases DAF-16 activity, resulting in extended mean lifespan in *C. elegans*. DAF-2 regulates LYS-7, which is encoded by an antibacterial gene, *lys-7* 15. A longevity-promoting factor, SKN-1, increases stress tolerance and extends lifespan in an IIS-dependent manner. SKN-1 is inhibited by DAF-2 in a DAF-16-independent manner^16^. Longevity is also affected by immune dysfunction (immunosenescence) in nematodes^17^. According to a previous report, probiotics (such as lactic acid bacteria), compared with a standard food (such as *E. coli* OP50), may enhance immunity and longevity in *C. elegans*^18^,^19^. Lactic acid bacteria, such as *Weissella koreensis* and *W. cibaria*, extend the lifespan of *C. elegans* and improve biomarkers of ageing, such as a reduction in lipofuscin accumulation, decreases in body size and enhancement of body movement^20^. Small body size is often associated with increased longevity and a well-known factor regulating body size is diet-restriction that induces longevity in *C. elegans*^21^. Diet-restricted worms exhibit small body size as well as increased lifespan. Although it has been reported that surface proteins and 1,4-dihydroxy-2-naphthoic acid from *P. freudenreichii* are involved in their anti-inflammatory effects^22^,^23^, little information, including the mechanisms of immunity and longevity in *C. elegans*, is available on SCFA-producing dairy bacteria. In this study, the effect of *P. freudenreichii* on the lifespan extension of *C. elegans* and enhancement of immune responses were investigated, and the mechanism underlying *P. freudenreichii*-mediated lifespan extension in *C. elegans* was elucidated using loss-of-function mutants.

## Results

### *P. freudenreichii* extends the mean lifespan of *C. elegans*

**Figure 1:**
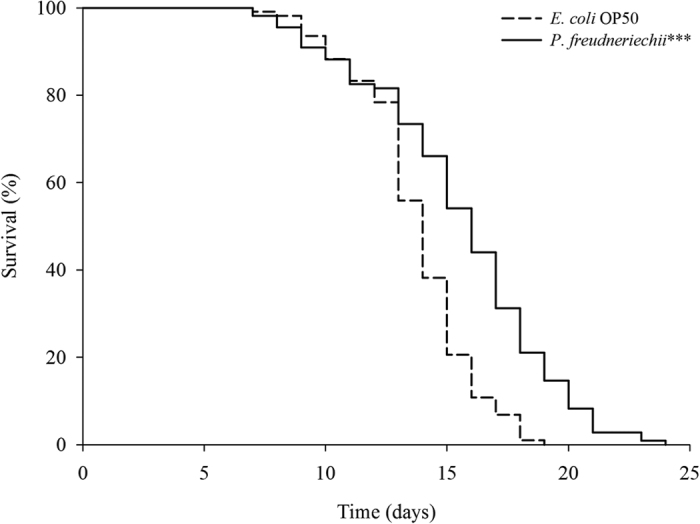
The effect of *P. freudenreichii* on the lifespan of *C. elegans* (N2). Maturing nematodes were fed *E. coli* OP50 until the L4 stage, and young adult worms were transferred to a fresh mNGM plate seeded with *E. coli* OP50 or *P. freudenreichii*. Significant differences shown are relative to *E. coli* OP50; ^***^p < 0.001.

*C. elegans* fed a lawn of *P. freudenreichii*, compared with the worms fed the standard *E. coli* OP50 lawn, showed a significantly (*p* < 0.001) increased mean lifespan (MLS) (Table 1). The MLS of *P. freudenreichii*-fed C*. elegans,* compared with that of *E. coli* OP50-fed worms, increased by approximately 13%. The survival rates were similar in both the *P. freudenreichii*- and *E. coli* OP50-fed C*. elegans* until day 13. After day 13, the two groups showed a significant difference in the survival rate (Fig. 1).

### The effects of *P. freudenreichii* on age-related biomarkers in *C. elegans*

**Figure 2:**
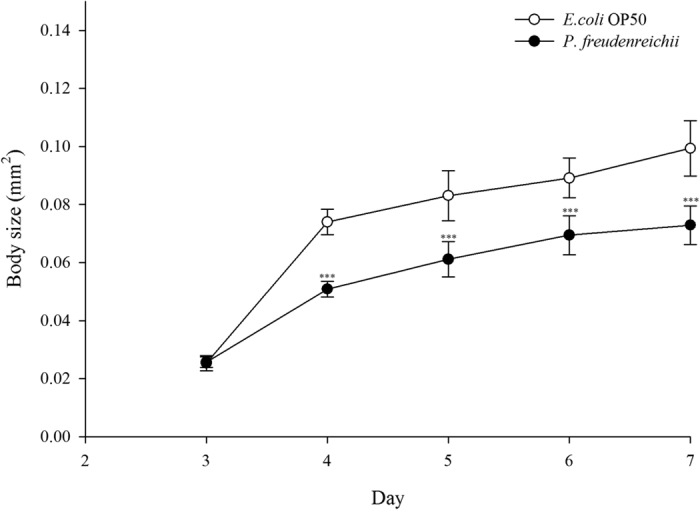
The effect of *P. freudenreichii* on *C. elegans* body size. Young adult nematodes were transferred to mNGM plates seeded with *P. freudenreichii* or *E. coli* OP50. Body size was determined with 12 worms for each bacterial strain. Significant differences shown are relative to *E. coli* OP50. The results are expressed as the mean ± standard deviation; ^***^p < 0.001.

Three-day-old worms with similar sizes were transferred to nematode growth medium (mNGM) plates seeded with *E. coli* OP50 or *P. freudenreichii*, and body size was measured for 4 days. Body size increased with age in all groups, and the *P. freudenreichii*-fed worms were significantly (*p* < 0.001) smaller than the *E. coli*-fed worms (Fig. 2).

**Figure 3:**
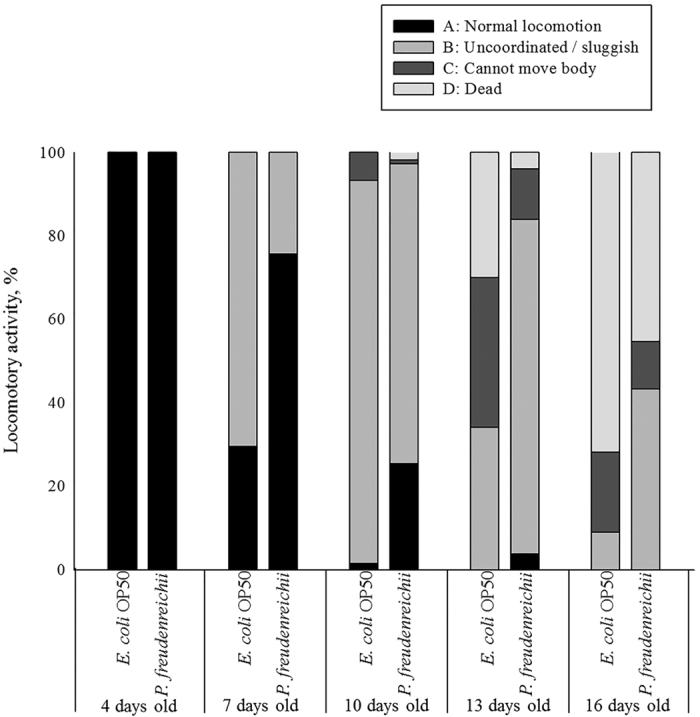
The locomotory activity of *C. elegans* (N2) fed *E. coli* OP50 or *P. freudenreichii*. Young adult worms fed *E. coli* OP50 for 3 days after hatching were transferred to fresh mNGM plates with 50 mg of *E. coli* OP50 or *P. freudenreichii* lawn. Individuals were assigned to four classes based on locomotion: class (**A**) normal coordinated sinusoidal locomotion; class (**B**) uncoordinated and/or sluggish movement; class (**C**) no movement except head or tail in response to prodding; and class (**D**) dead worms. The bars indicate the proportion of each class at the designated time.

Body movement is related to age and the gut muscle. As the nematodes age, body movement becomes blunter and weaker. The locomotory rate of *C. elegans* on days 4, 7, 10, 13 and 16 was measured. During the experimental period, the proportion of worms displaying sinusoidal locomotion (class A) was always higher in *P. freudenreichii*-fed *C. elegans* than in *E. coli* OP50-fed worms (Fig. 3).

**Figure 4:**
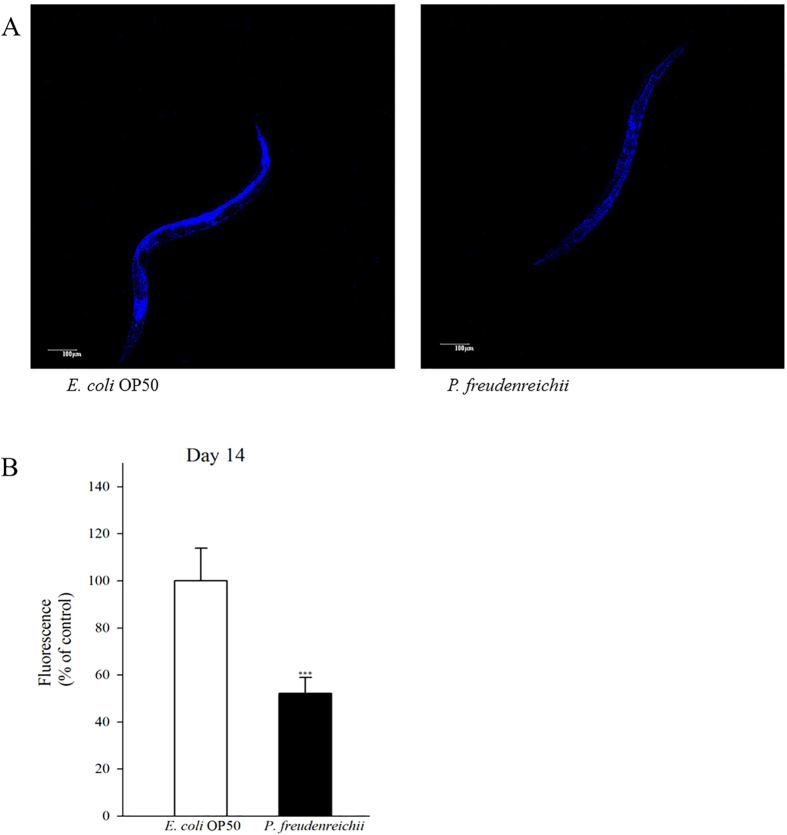
Lipofuscin accumulation in *C. elegans* (N2) fed *E. coli* OP50 or *P. freudenreichii*. L4 stage worms were transferred to the mNGM plates with *E. coli* OP50 or *P. freudenreichii* and fed bacteria for 14 days. After 14 days, lipofuscin was measured by assessing autofluorescence using a confocal microscope. (**A**) Fluorescence of lipofuscin in worms fed *E. coli* OP50 and *P. freudenreichii* on day 14. (**B**) Fluorescence was quantified using ImageJ software. The graph depicts the mean percentage in arbitrary units relative to that of control worms fed *E. coli* OP50 on day 14; ^***^p < 0.001. Scale bar = 100 μm.

Lipofuscin accumulation is a biomarker of ageing in *C. elegans*. The accumulation of lipid granules was determined by autofluorescence. The intensity of 4′,6-diamidino-2-phenylindole (DAPI) decreases when nematode ageing is delayed^18^,^24^. The autofluorescence of worms fed *P. freudenreichii* was significantly (*p* < 0.001) decreased compared with that of worms fed *E. coli* OP50 (Fig. 4A). The lipofuscin level in *P. freudenreichii*-fed worms was approximately 50% of the level observed in *E. coli* OP50-fed worms (Fig. 4B).

### Expression of lifespan extension-related genes

**Figure 5:**
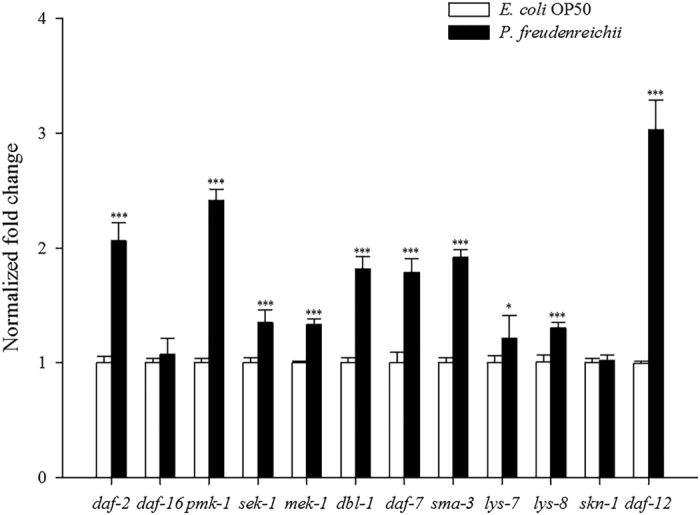
The expression levels of genes related to lifespan extension and immune response in *C. elegans.* The experiment was independently performed three times. Significant differences shown are relative to *E. coli* OP50. The expression level of each gene was normalized to that of *act-1*. The results are shown as the mean ± standard deviation (^*^p < 0.05, ^***^p < 0.001).

Quantitative real-time polymerase chain reaction (qPCR) analysis was conducted to investigate the expression of genes related to lifespan extension and immune response in *C. elegans* fed *E. coli* OP50 or *P. freudenreichii*. The expression levels of the *daf-2, daf-16, pmk-1, sek-1, mek-1, dbl-1, daf-7, sma-3, skn-1* and *daf-12* genes were measured. All genes except *daf-16* and *skn-1* were significantly up-regulated (*p* < 0.05) (Fig. 5). All genes involved in the p38 MAPK pathway and the TGF-β pathway assessed in this study were significantly up-regulated (*p* < 0.05). Notably, *daf-12* expression increased 3.1-fold compared with that in the control. In addition, *lys-7* and *lys-8*, antimicrobial peptide genes, were significantly overexpressed (*p* < 0.05).

### Lifespan extension in *C. elegans* mutants

To determine whether MLS extension is related to enhanced innate immunity, *C. elegans* loss-of-function mutants for genes related to innate immunity and lifespan extension were fed *P. freudenreichii* or *E. coli* OP50, and their longevity was measured. The mutants defective in innate immunity were selected based on previous studies^13^,^14^,^25^ and our qPCR results (Fig. 5). *P. freudenreichii* failed to extend the lifespan of the *pmk-1, sek-1* and *mek-1* mutants, which are defective in genes involved in the p38 MAPK pathway; the *dbl-1* mutant, which is defective in a gene involved in the TGF-β pathway; and the *daf-2* mutant, which contains a mutation in the insulin/IGF receptor orthologue (Table 1, Fig. S1). However, the *jnk-1, daf-16, skn-1* and *daf-7* mutants had a significantly extended MLS after feeding on *P. freudenreichii (p* < 0.05). Interestingly, the *daf-12* mutant, which is defective for a gene encoding a nuclear hormone receptor that acts as a lipid transcription factor activated by steroids and fatty acids^26^, had a significantly decreased MLS (*p* < 0.001). The results suggested that *P. freudenreichii* may activate the p38 MAPK and TGF-β pathways, which are related to innate immunity and lifespan extension.

### Food preference of *C. elegans*

To confirm that the lifespan extension effect of *P. freudenreichii* was not derived from a preference between *P. freudenreichii* and *E. coli* OP50, binary choice assay was carried out. *C. elegans* showed positive food preference (CI =  +0.13) for *P. freudenreichii*, which means that *C. elegans* did not show any preference between *E. coli* OP50 and *P. freudenreichii* (Fig. S2). The results suggest that the lifespan extension effect of *P. freudenreichii* is not due to dietary restriction.

### *C. elegans* fed *P. freudenreichii* exhibits resistance to *Salmonella typhimurium*

**Figure 6:**
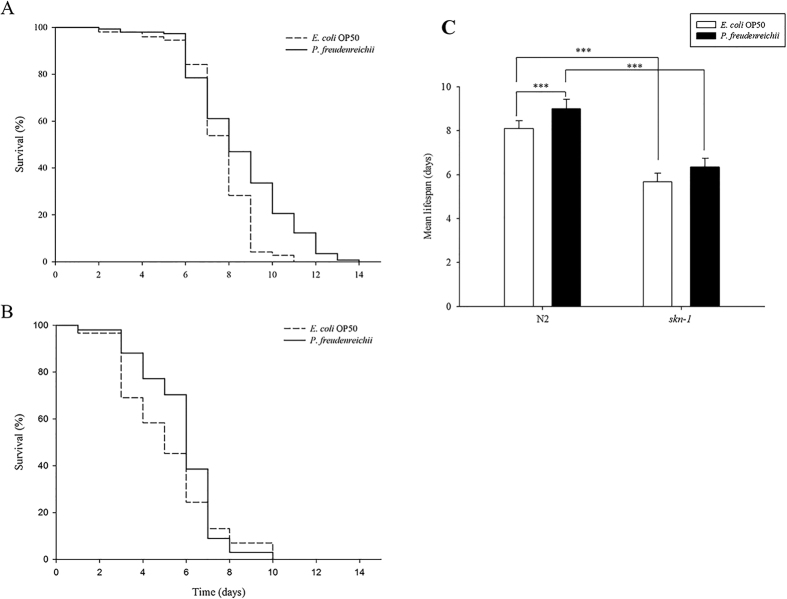
The effect of *P. freudenreichii* on resistance against *S. typhimurium* in *C. elegans*. (**A**) L4 stage *C. elegans* (N2) and (**B**) *skn-1* mutants were fed *E. coli* OP50 or *P. freudenreichii* for 4 days and transferred to fresh NGM/FUdR seeded with *S. typhimurium.* The survival rate was measured every 24 h after transferring the worms to the plate seeded with the pathogen. (**C**) Mean lifespan of *C. elegans* (N2) and *skn-1* mutants infected with *S. typhimurium*. All experiments were performed at least three times; ^***^p < 0.001.

When worms are exposed to pathogens, the *skn-1* gene is expressed via the activation of the PMK-1 pathway^27^. To determine whether the resistance of worms to pathogenic bacteria is enhanced, *C. elegans* wild type (N2) and the *skn-1* mutant were exposed to *S. typhimurium*, and the survival rate was measured. Compared to the *skn-1* mutant, *C. elegans* wild type (N2) worms exhibited increased resistance to *S. typhimurium (p* < 0.001) (Fig. 6A). The survival rates of *C. elegans* were similar until day 7, but after day 7, *P. freudenreichii*-fed worms had a higher survival rate than *E. coli* OP50-fed worms. However, the *skn-1* mutant failed to show an extended MLS when fed *P. freudenreichii* (Fig. 6B). The MLS of *P. freudenreichii*-fed *C. elegans* (N2), compared with that of the *E. coli* OP50-fed worms, increased by approximately 11% (Fig. 6C). The MLS decreased approximately 30% in both *E. coli* OP50- and *P. freudenreichii*-fed *skn-1* mutants compared with *C. elegans* (N2). The results suggested that *P. freudenreichii* activates the defence system of *C. elegans* during infection via the p38 MAPK pathway.

## Discussion

Several factors, such as body size, lipofuscin and locomotory activity, change during ageing^13^,^18^,^24^, and thus, they are used as biomarkers of ageing. Feeding *P. freudenreichii,* compared *E. coli* OP50, to *C. elegans* reduced lipofuscin accumulation, decreased body size and increased locomotory activity, which suggests that *P. freudenreichii* extends the MLS of *C. elegans*. Additionally, compared with *E. coli* OP50-fed worms, *P. freudenreichii*-fed *C. elegans* showed a significantly increased MLS.

Genes related to immunity, neuroendocrine signalling, dietary restriction and mitochondrial function modulate the lifespan of *C. elegans*^28^. The results of food preference test suggest that dietary restriction is not linked to the lifespan extension of *P. freudenreichii*-fed *C. elegans. C. elegans* has a deficient adaptive immunity system and an NF-κB-based defence system; nematodes are dependent on the innate immune system for immune responses^29^. TGF-β signalling plays an important role in the regulation of cell growth, differentiation and development in many biological systems. The TGF-β pathway is composed of the DBL-1/SMA pathway and the DAF-7 pathway. The DBL-1 ligand is related to innate immunity and body morphology in *C. elegans*, while DAF-7 is the TGF-β-related ligand for the dauer pathway. *P. freudenreichii* did not extend the MLS of *dbl-1* mutants, but it increased the MLS of *daf-7* mutants. SMA-3 plays in a role in innate immunity^30^. The expression level of the *sma-3* gene, which is located downstream of *dbl-1*, significantly increased. The results suggest that *P. freudenreichii* affects the MLS of *C. elegans* via the TGF-β pathway by activating DBL-1 but not by activating DAF-7. *lys-8*, a broad-spectrum antimicrobial peptide, encodes a lysozyme and is regulated by the DBL-1 pathway^25^. Lysozyme plays an important role in innate immunity and acts directly in the nematode defence system against infection^25^. In this study, *P. freudenreichii* influenced the expression of the *lys-8* gene. Feeding *P. freudenreichii* to *C. elegans* up-regulated infection-inducible genes, such as *sma-3* and *lys-8*, under the control of the DBL-1/TGF-β pathway, which may increase the resistance to infection in *C. elegans*.

p38 mitogen-activated protein kinase (MAPK) is involved in innate immunity in *C. elegans* and influences lifespan extension in *C. elegans*^31^. *pmk-1, sek-1, mek-1* and *skn-1* are components of the MAPK pathway in *C. elegans*. The *pmk-1, sek-1* and *mek-1* mutants failed to show increases in MLS of nematodes fed *P. freudenreichii* compared with worms fed *E. coli* OP50. The p38 MAPK pathway in *C. elegans* is similar to the mammalian p38 MAPK pathway that regulates the production of antimicrobials and inflammatory cytokines in response to lipopolysaccharide^31^. SKN-1, a transcription factor in the oxidative stress response, is phosphorylated by the p38 MAPK orthologue PMK-1 and is expressed during pathogen exposure^27^,^32^. *skn-1* mutants fed *P. freudenreichii* had an increased MLS. However, interestingly, when exposed to a pathogen, *P. freudenreichii* feeding, compared with *E. coli* OP50 feeding, increased the survival of *C. elegans* (N2); however, the lifespans of *P. freudenreichii-* and *E. coli* OP50*-*fed *skn-1* mutants decreased significantly compared with that of *C. elegans* (N2), without a significant difference between them. These results suggest that *P. freudenreichii* activates the p38 MAPK pathway transcription factor *skn-1* during pathogenic infection in *C. elegans*. Although SKN-1 did not affect the longevity of *C. elegans* mediated by *P. freudenreichii* in normal conditions, SKN-1 might play an important role in pathogen resistance and survival during infection.

DAF-2 is the upstream receptor of the IIS signal and controls many downstream genes that contribute to nematode survival^13^. DAF-16, an orthologue of the FOXO transcription factor and the IIS transcription factor, is also a key player in innate immunity in *C. elegans*^33^. The *daf-16* mutant shows no resistance to pathogens^13^. *daf-16* acts in the insulin/IGF-1-mediated signalling pathway that regulates longevity in dietary restricted conditions, detoxification and oxidative stress. The *daf-16* mutant showed significantly increased MLS of worms fed *P. freudenreichii* compared with worms fed *E. coli* OP50, and the expression of the *daf-16* gene in *P. freudenreichii*-fed *C. elegans,* compared with that in *E. coli* OP50-fed worms, did not significantly increase. Mutation in *daf-2* activated DAF-16, resulting in the extension of lifespan in *C. elegans*. In this study, the *daf-2* mutant did not prolong lifespan, but the *daf-16* mutant did extend the MLS of *C. elegans*, indicating that the longevity effect of *P. freudenreichii* is not related to the daf-2/daf-16 pathway. The results suggest that *P. freudenreichii* influences *C. elegans* innate immunity through the TGF-β and p38 MAPK pathways but not through the IIS pathway.

*lys-7* encodes an *enzyme* homologous to an antimicrobial lysozyme that functions in the innate immune response, indicating that *lys-7* is involved in pathogen resistance^31^. *lys-7* is a DAF-16-dependent gene and is down-regulated by DAF-2. Curiously, in this study, although *P. freudenreichii* did not stimulate the expression of *daf-16* and induced *daf-2* expression, *lys-7* was significantly (*p* < 0.05) expressed in worms fed *P. freudenreichii* compared with worms fed *E. coli* OP50. DAF16-independent alternate pathway(s) may contribute to *lys-7* expression in worms fed *P. freudenreichii*.

DAF-12 is a well-known nuclear hormone receptor and affects the innate immune system by inducing the production of antimicrobial peptides. DAF-12 is correlated with the p38 MAPK pathway^34^,^35^. *daf-12* directly regulates *skn-1*, a component of the PMK-1 immune signalling pathway, during pathogen infection. It was reported that DAF-12 is involved in the innate immune system via crosstalk with the p38/PMK-1 MAPK pathway^34^. However, a clear relationship between *pmk-1* and *daf-12* has not been elucidated. In this study, we found that *pmk-1* and *daf-12* mutants failed to extend the MLS of *P. freudenreichii*-fed *C. elegans*, while the *skn-1* mutant extended the MLS of *P. freudenreichii*-fed worms. These results indicate that there may be an alternative pathway between PMK-1 and DAF-12 that does not involve SKN-1 implicated in the MLS extension of *C. elegans* by *P. freudenreichii. daf-12* is a putative target gene of DAF-7/TGF-β (dauer pathway); however, DAF-12 might act in a DAF-7-independent manner to alter longevity in *P. freudenreichii*-fed *C. elegans*. In addition, DAF-12 plays an important role in longevity through the integration of both germline and DAF-2 signalling^36^. DAF-12, similar to DAF-16, is negatively regulated by DAF-2. However, in this study, DAF-12, in contrast to DAF-16, affected the longevity of *P. freudenreichii*-fed *C. elegans*. The results suggest that DAF-12 is involved in the longevity of *P. freudenreichii*-fed *C. elegans* through an alternative pathway in a DAF-2- and DAF-7-independent manner.

**Figure 7:**
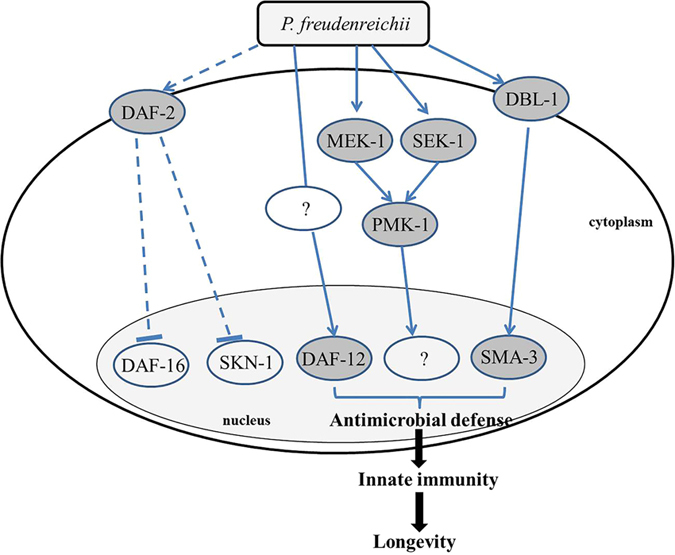
Mechanism predicted to be involved in the lifespan extension effect of *P. freudenreichii* in *C. elegans.* DAF-16, the primary transcription factor required for lifespan extension, is not related to lifespan extension of *P. freudenreichii*-fed *C. elegans*. DBL-1 activates SMA*-*3 through the TGF-β pathway, which produces antimicrobial peptides. MEK-1, SEK-1 and PMK-1, the components of the MAPK pathway, are regulated to extend lifespan in *P. freudenreichii*-fed *C. elegans*. A nuclear hormone receptor, DAF-12, is also activated by *P. freudenreichii* and is involved in the extension of lifespan in *C. elegans*. The solid lines represent potential pathways and putative proteins activated by *P. freudenreichii* are shown in a dark circle, and dashed lines indicate speculated pathways that are not involved in the lifespan extension in *P. freudenreichii*-fed *C. elegans*.

Based on the gene expression and mutant survival data obtained in this study, we developed a model of the pathways involved in the MLS extension of *P. freudenreichii*-fed *C. elegans* (Fig. 7). The molecular mechanisms that modulate ageing and immune response are linked in *C. elegans*. DAF-2–mediated insulin signalling pathway is universal in regulating ageing and p38 MAPK regulates the innate immune function in *C. elegans*, which are conserved among living organisms including human^13^. Therefore, it is possible that the mechanisms identified in this study may apply to other species including humans. In conclusion, dairy *P. freudenreichii* extends the MLS of *C. elegans* by activating the innate immune system via the p38 MAPK pathway- and the TGF-β pathway-dependent but IIS pathway-independent manner.

## Methods

### Bacteria culture conditions and strains

*P. freudenreichii* KCTC 1063 was obtained from the Korean Collection for Type Cultures (KCTC), and *Escherichia coli* OP50 was provided by the Caenorhabditis Genetics Centre (CGC) at the University of Minnesota and used as a standard food for *C. elegans. E. coli* OP50 was grown in Luria-Bertani (LB) broth (Difco, Detroit, MI, USA) at 37 °C with shaking overnight. *P. freudenreichii* was cultured using reinforced clostridial medium (RCM) broth (Oxoid, Hampshire, United Kingdom) for 48–72 h in anaerobic conditions using a BD GasPak (Becton Dickinson, Franklin Lakes, NJ, USA) at 30 °C. *E. coli* OP50 and *P. freudenreichii* were collected by centrifugation and washed twice with M9 buffer. Then, the bacteria were diluted to a final concentration of 0.1* *mg (wet weight) per mL in M9 buffer^37^.

### Nematode preparation

*C. elegans* Bristol strain N2 was used as the wild-type strain, and the mutant strains were provided by CGC. The mutants used in this study were CF1038 *daf-16* (mu86), CB1370 *daf-2* (e1370), EU1 *skn-1* (zu67), VC8 *jnk-1* (gk7), FK171 *mek-1* (ks54), KU25 *pmk-1* (km25), KU4 *sek-1* (km4), RB2302 *daf*-7 (ok3125), NU3 *dbl-1* (nk3) and DR20 *daf-12* (m20). Worms were maintained and propagated in peptone-free mNGM (modified nematode growth medium) at 25 °C using standard techniques^38^. Some bacteria consume media components, which may affect nematode survival^18^. *E. coli* OP50 and *P. freudenreichii* suspended in M9 buffer were seeded on mNGM in 90 mm diameter petri dishes to feed the worms. *C. elegans* was exposed to a sodium hypochlorite-sodium hydroxide solution as previously described to obtain viable eggs^39^. The eggs were incubated overnight in M9 buffer at 25 °C for hatching, and the suspension of L1-stage worms was centrifuged at 1,200 × g for 2* *min. After eliminating the supernatant, the remaining larvae were transferred to fresh mNGM plates seeded with *E. coli* OP50 and incubated at 25 °C. The worms were allowed to grow on *E. coli* OP50 until the L4 stage^18^. All experiments used 3-day-old worms (1 day adult) to regulate the reproductive system in *C. elegans*, with the exception of the mutants^40^.

### Determination of the mean lifespan of *C. elegans*

The *C. elegans* lifespan assay was performed by transferring 15 young adult (L4 stage) worms per bacterial species to three mNGM/FUdR plates. 5-Fluoro-2′-deoxyuridine (FUdR, Sigma Aldrich, St. Louis, MO, USA) (50* *μM) was added to the plates^41^, and *E. coli* OP50 and *P. freudenreichii* were seeded on them. The plates were incubated at 25 °C, and the numbers of live or dead worms were scored every 24* *h. Worms were considered “dead” when they failed to respond to a gentle touch with a worm picker. Nematodes that crawled off the plates and showed non-natural deaths, such as bagging or adhering to the wall of the plate, were censored^42^. For the first 3 days, worms were transferred to fresh mNGM/FUdR every day, and then they were transferred once per week for the rest of the experiment to maintain a sufficient food source. Experiments were performed at least in triplicate, and more than 100 worms for each bacterial species were used in the longevity assay.

The mean lifespan was estimated using the following formula^43^:


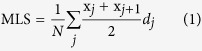


where *j* is the age category (day), *dj* is the number of worms that died in the age interval (xj, xj1), and *n* is the total number of worms. The standard error (SE) of the MLS estimate was calculated using the following equation:


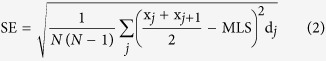


### Measurement of body size

Young adult worms (L4 stage) were transferred to mNGM plates (60 mm plate) covered with 5 mg (wet weight) of *E. coli* OP50 or *P. freudenreichii* cells in M9 buffer. The plates were incubated at 25 °C, and the body size of the live worms was measured every 24 h until 7 days of age. In total, 12 worms per bacterial species were measured. Images of *C. elegans* were taken with a stereomicroscope (Olympus SZ61) and a ToupCam (UCMOS05100KPA), and the images were analysed using ToupCam software. For this experiment, the area of the worm’s projection was estimated automatically and used as an index of body size^19^. Three independent experiments were performed for each bacterial species.

### Lipofuscin accumulation

The autofluorescence of intestinal lipofuscin was measured as an index of senescence of day 14 adults. Randomly selected worms from each bacterial lawn were washed three times with M9 buffer. Then, the worms were placed onto a 5% agar pad coated with 10 mM sodium azide in M9 buffer to be paralyzed. Lipofuscin autofluorescence images were taken using blue excitation light (405**−**488 nm) with DAPI (4′,6-diamidino-2-phenylindole) and laser confocal scanning microscopy (Olympus Ix81-FV1000, Tokyo, Japan)^18^. Fluorescence was quantified using ImageJ software (National Institutes of Health, Bethesda, MD, USA) to determine the lipofuscin levels. Three independent experiments were performed with more than 30 worms for each bacterial species on each day.

### Locomotory scoring

The locomotory assay of worms at different ages was performed using a scoring method described in previous reports^18^. Nematodes were classified as class “A” when they showed spontaneous movement or vigorous locomotion in response to prodding, class “B” worms did not move unless touched or appeared to have uncoordinated movement, class “C” worms moved only their head and/or tail in response to prodding, and class “D” worms were dead. Experiments were conducted at least three times (n > 100) independently for each bacterial species.

### Binary choice assay

To examine the preference of worms towards *P. freudenreichii*, we conducted binary choice assay compared with *E. coli* OP50 44. Assays were done on NGM medium in 90 mm plates. Bacterial food was seeded at the same distance from the sides of the plates with a diameter of 0.5 cm (Fig. S3), and L4 stage nematodes (30 worms) were put on the centre of the plates. The number of worms in each bacterial lawn was counted after 3 h of incubation at 25 °C. Three independent experiments were performed. The choice index (CI) was calculated as follows:


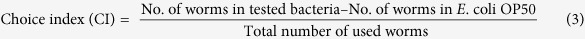


If, CI = −1.0 represents complete preference for *E. coli* OP50. +1.0 represents complete preference for the test bacterium.

0.0 represents an equal distribution.

### Resistance against *Salmonella* infection

After hatching, the nematodes were grown on *E. coli* OP50 until they were 3-day-old adults. Then, the worms were allocated to a control group that continued to be fed *E. coli* OP50 or the test group that received *P. freudenreichii* for 4 days. The 7-day-old worms were transferred to NGM agar plates with *Salmonella typhimurium* ATCC 13311 cell suspensions (concentration of 0.1 mg/mL). The bacterial suspensions were incubated at room temperature for 12 h before the nematodes were transferred to the NGM plates^45^. Then, 45 worms from each bacterial group were divided into three plates that were seeded with *Salmonella* and incubated at 25 °C. The numbers of live and dead nematodes were scored every 24 h. The survival rate of *C. elegans* exposed to *Salmonella* was compared with that of worms without *Salmonella*^19^. Each test was performed with at least 100 worms.

### RNA isolation and qPCR

Worms fed *E. coli* OP50 or *P. freudenreichii* for 24 h were collected and washed twice with sterilized M9 buffer. Then, total mRNA from whole worms was isolated using TRIzol as previously described^46^. Total RNA was converted to cDNA using a RevertAid First Strand cDNA Synthesis Kit according to the manufacturer’s instructions (Thermo Scientific, Wilmington, DE, USA), followed by qPCR using SYBR Green (KAPA Biosystems, Wilmington, MA, USA) and a QuantStudio 6 Flex Real Time PCR machine (Applied Biosystems, Foster City, CA, USA). The primer sequences are listed in Table S1. The reactions had an initial step at 95 °C, followed by 40 cycles of 95 °C for 20 s, 60 °C for 20 s and 72 °C for 30 s, followed by melt curve analysis. The experiments were independently performed three times, and relative expression levels were calculated using the 2^−ΔΔCT^ method^46^. The control gene *act-1* was used to normalize gene expression data.

### Statistical analysis

The MLS of *C. elegans* was calculated using the Kaplan-Meier method, and the *p*-value of survival differences was tested using the log-rank test^37^. In other experiments, the significance of comparisons between *E. coli* OP50 and *P. freudenreichii* was determined using Student’s *t*-test. Significance was defined as a *p*-value under 0.05 in all experiments. If the data were not normally distributed, the Mann-Whitney U test was used^18^.

## Additional Information

**How to cite this article**: Kwon, G. *et al*. Dairy *Propionibacterium* extends the mean lifespan of *Caenorhabditis elegans* via activation of the innate immune system. *Sci. Rep.* **6**, 31713; doi: 10.1038/srep31713 (2016).

## Supplementary Material

Supplementary Information

## Figures and Tables

**Table 1:** The mean lifespan of *C. elegans* (N2) and loss-of-function mutants fed *E. coli* OP50 and *P. freudenreichii*.

Nematode type	Food source (bacteria)	MLS ± SE (days) (n)
N2 (wild type)	*E. coli* OP50	14.24 ± 0.77 (102)
	*P. freudenreichii*	16.06 ± 0.92^***^ (109)
KU25 *pmk-1* (km25)	*E. coli* OP50	14.49 ± 0.79 (119)
	*P. freudenreichii*	14.84 ± 0.87 (109)
KU4 *sek-1* (km4)	*E. coli* OP50	12.24 ± 0.71 (120)
	*P. freudenreichii*	12.56 ± 0.78 (109)
FK171 *mek-1* (ks54)	*E. coli* OP50	11.18 ± 0.78 (103)
	*P. freudenreichii*	11.40 ± 0.77 (115)
NU3 *dbl-1* (nk3)	*E. coli* OP50	11.87 ± 0.69 (108)
	*P. freudenreichii*	12.44 ± 0.71 (101)
CB1370 *daf-2* (e1370)	*E. coli* OP50	31.42 ± 1.82 (162)
	*P. freudenreichii*	31.98 ± 2.62 (100)
DR20 *daf-12* (m20)	*E. coli* OP50	14.33 ± 0.79 (128)
	*P. freudenreichii*	13.55 ± 0.79^***^ (108)
VC8 *jnk-1* (gk7)	*E. coli* OP50	13.56 ± 0.69 (124)
	*P. freudenreichii*	15.05 ± 0.82^***^ (113)
CF1038 *daf-16* (mu86)	*E. coli* OP50	11.81 ± 0.55 (137)
	*P. freudenreichii*	12.85 ± 0.64^***^ (124)
EU1 *skn-1* (zu67)	*E. coli* OP50	12.85 ± 0.68 (125)
	*P. freudenreichii*	14.24 ± 0.80^**^ (107)
RB2302 *daf-7* (ok3125)	*E. coli OP50*	18.57 ± 1.33 (104)
	*P. freudenreichii*	26.51 ± 1.82^***^ (106)

n, Number of total live worms; *p*-values were calculated relative to controls (*E. coli* OP50); ^**^p < 0.01, ^***^p < 0.001.
