# Supplementary material for: Dairy Propionibacterium extends the mean lifespan of Caenorhabditis elegans via activation of the innate immune system
Source: Sci Rep. 2016 Aug 17;6:31713. doi: 10.1038/srep31713 (PMC4987649; doi:10.1038/srep31713)
Supplement: Supplementary Information [file srep31713-s1.pdf]

Supplementary Information

Dairy *Propionibacterium* extends the mean lifespan of *Caenorhabditis elegans* via activation of the innate immune system

Gayeung Kwon, Jiyun Lee, Young-Hee Lim

Table S1. Primer sequences used in this study.

Gene		Primer sequence	Product size (bp)
act-1	Forward	5' CACGGTATCGTCACCAACTG 3'	122
	Reverse	5' GCTTCAGTGAGGAGGACTGG 3'	
daf-2	Forward	5' GCCCGAATGTTGTGAAAACT 3'	185
	Reverse	5' CCAGTGCTTCTGAATCGTCA 3'	
daf-16	Forward	5' TCCTCATTCACTCCCGATTC 3'	175
	Reverse	5' CCGGTATATTCATGAACGTG 3'	
pmk-1	Forward	5' ACTCGCCGTGATTTCAAACG 3'	100
	Reverse	5' CAGTTGGACGACGATCTGGA 3'	
sek-1	Forward	5' TGGCAAACACATTCCAGAGC 3'	196
	Reverse	5' AGTCTTGGCCATGCTGTTTG 3'	
mek-1	Forward	5' GCCTCATCATCCATCCCGTA 3'	135
	Reverse	5' TCCACGAACTGAAGCTCCTT 3'	
dbl-1	Forward	5' GCCATTCTCCACCTCTTCCT 3'	149
	Reverse	5' GGAACATCAATGCTCGGACC 3'	
daf-7	Forward	5' GTGCTGCTTGTATGACCTCG 3'	136
	Reverse	5' GGTTTCCGCCAAGTTGAAGT 3'	
lys-7	Forward	5' ATTCAGGTCACTTCGCCAAC 3'	148
	Reverse	5' ATCCGGTCGTGATCTGATTC 3'	
lys-8	Forward	5' CTCCACGAGTTCCACCAAAT 3'	124
	Reverse	5' TATGCACGGACAAAGACTGC 3'	
sma-3	Forward	5' CCACCGCACAGTCAATTATG 3'	144
	Reverse	5' ATCTTCTCCCCAGCCCTTTA 3'	
daf-12	Forward	5' GTTCTGGTGAAGCCGAAGAG 3'	178
	Reverse	5' AAGGGTGGTTGAGGTACGTG 3'	
skn-1	Forward	5' TCAGGACGTCAACAGCAGAC 3'	164
	Reverse	5' CGTGGAGATTCCGAAGAGAG 3'	

A

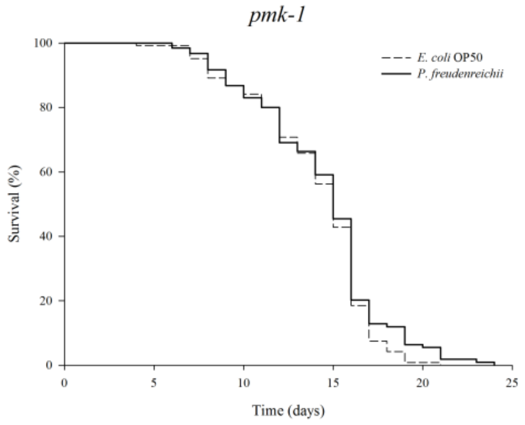
pmk-1
E. coli OP50
P. freudenreichii
Survival (%)
Time (days)

B

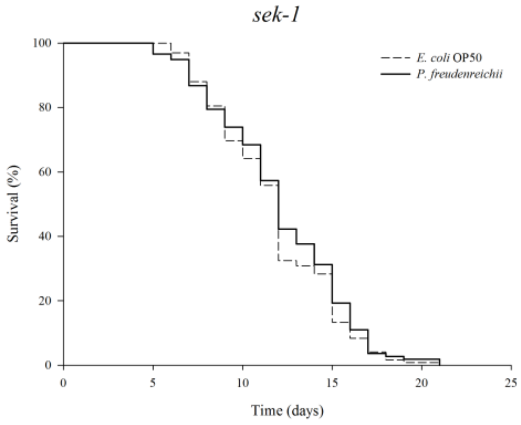
sek-1
E. coli OP50
P. freudenreichii
Survival (%)
Time (days)

C

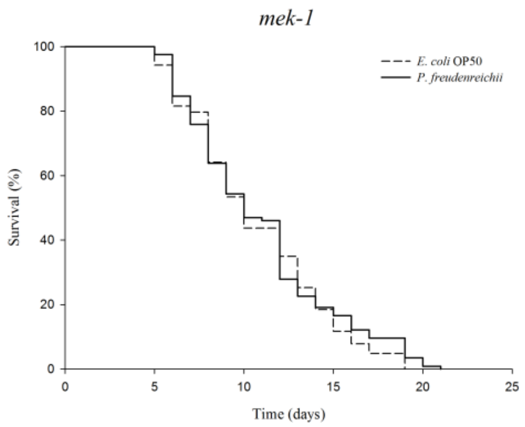
mek-1
E. coli OP50
P. freudenreichii
Survival (%)
Time (days)

D

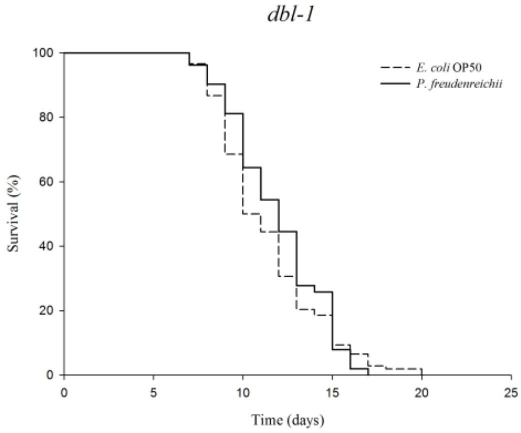
dbl-1
E. coli OP50
P. freudenreichii
Survival (%)
Time (days)

E

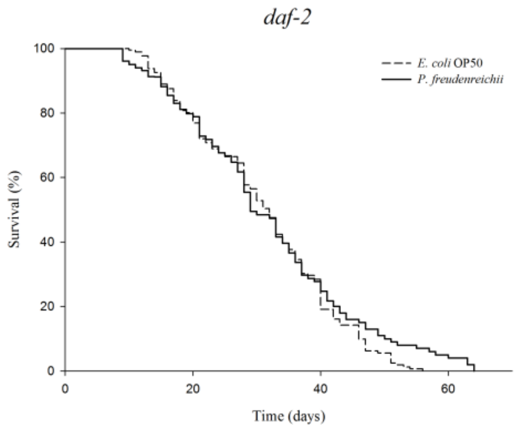
daf-2
E. coli OP50
P. freudenreichii
Survival (%)
Time (days)

F

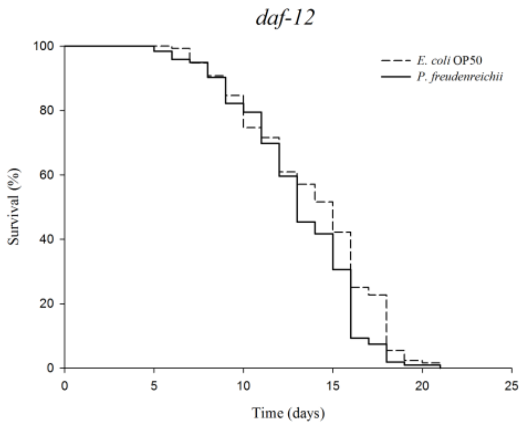
daf-12
E. coli OP50
P. freudenreichii
Survival (%)
Time (days)

G

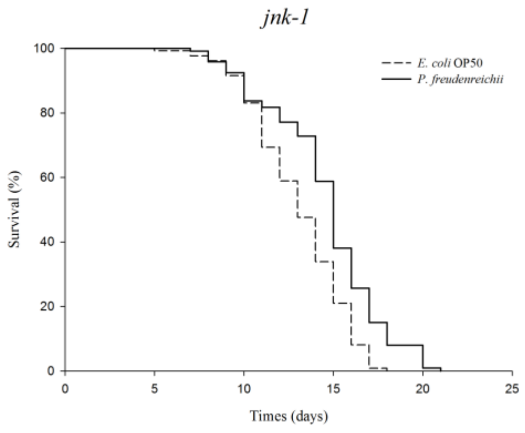

H

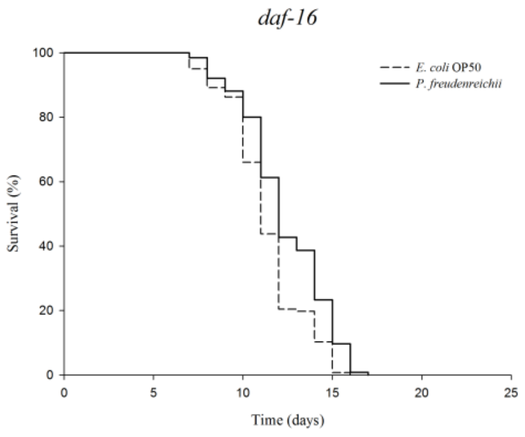

I

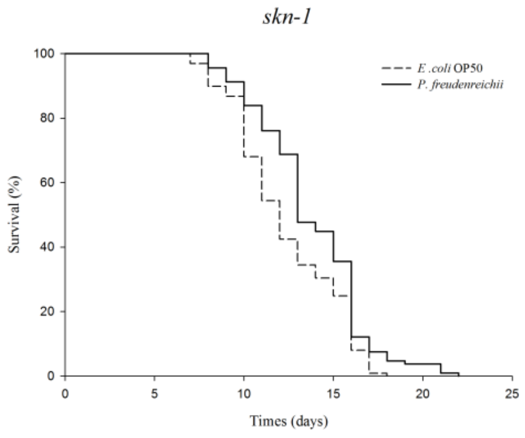

J

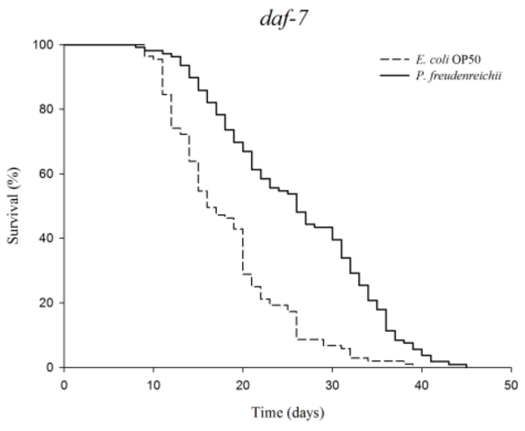

Figure S1. Effect of *P. freudenreichii* on the lifespan of *C. elegans* loss-of-function mutants. *p*-values were calculated using the log-rank test. Data were compared with *E. coli* OP50; $^{**}p < 0.01$, $^{***}p < 0.001$.

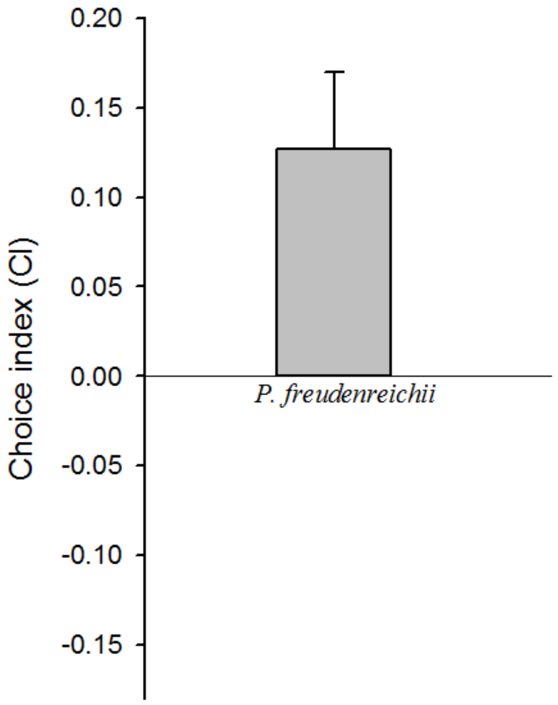

Figure S2. Binary choice assay between *P. freudenreichii* and *E. coli* OP50. The food choice index of *P. freudenreichii* compared with *E. coli* OP50.

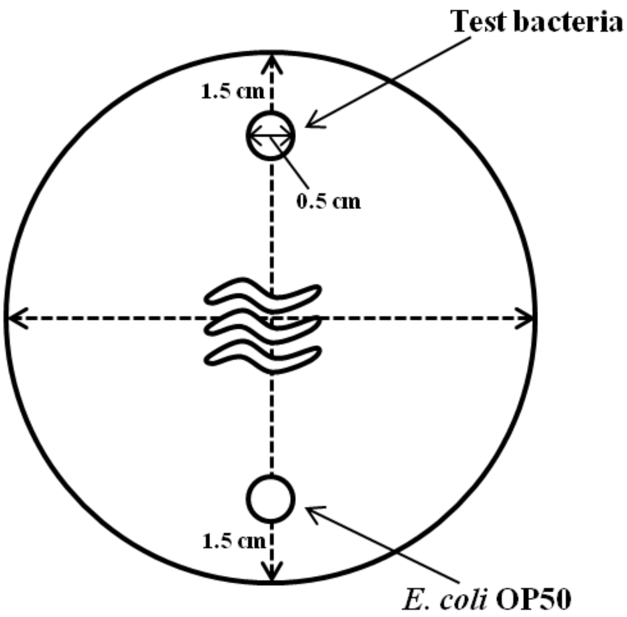

Figure S3. Schematic representation of plates used for the binary choice assay. Bacterial food was seeded at the same distance from the sides of the plates with a diameter of 0.5 cm, and nematodes (30 worms) were put on the centre of the plates.
